# Diagnosis of Endometriosis by Transvaginal Ultrasound: An Online Survey of Gynecologists Practising in Greece

**DOI:** 10.7759/cureus.37950

**Published:** 2023-04-21

**Authors:** Georgios Grigoriadis, Horace Roman, Dimitrios R Kalaitzopoulos, Nikos Christoforidis, George Pados, Angelos Daniilidis

**Affiliations:** 1 Department of Obstetrics and Gynecology, Aristotle University of Thessaloniki, Thessaloniki, GRC; 2 Department of Endometriosis Surgery, Franco-European Multidisciplinary Institute for Endometriosis, Bordeaux, FRA; 3 Department of Obstetrics and Gynecology, Cantonal Hospital Schaffhausen, Schaffhausen, CHE; 4 Fertility Unit, Embryolab, Thessaloniki, GRC

**Keywords:** endometrioma, survey, diagnosis, ultrasound, endometriosis

## Abstract

Accurate diagnosis and assessment of endometriosis by transvaginal ultrasound scan (TVS) can be challenging. We performed an online survey of specialist gynecologists who perform TVS on a regular basis regarding their views as well as clinical experience on the use of TVS in the diagnosis of endometrioma and deep endometriosis (DE). We collected 64 responses. Sixty-one participants (95.31%) answered that they can confidently diagnose endometrioma by TVS "always" or "most of the time". With the exception of DE of the recto-vaginal septum/posterior vaginal vault, for all other DE locations, more than 50% of participants felt that they can "rarely" or "never" diagnose it by TVS in their own clinical practice. Forty-two participants (65.6%) stated that additional, specialized training is required for the diagnosis of endometrioma. When asked about a diagnosis of DE, 58 participants (90.6%) felt that the same is required. The only statistically significant association was between the number of TVSs performed per year and the clinician's ability to diagnose bowel DE in their practice. The answers to all other questions did not differ significantly based on professional status, years of experience post-residency, or number of TVSs per year. Our results demonstrate the delayed adoption of novel diagnostic approaches in endometriosis and confirm the urgent need for specialized ultrasound training.

## Introduction

Endometriosis is a common gynecological disease, affecting 5-10% of women of reproductive age [1]. It is characterized by the presence of ectopic endometrial glands and stroma outside the uterus [2]. The disease is commonly associated with pelvic pain and infertility. Endometriosis is a heterogeneous disease, and three phenotypes are commonly recognized: superficial peritoneal lesions (SUP), ovarian endometriomas (OMA), and deep endometriosis (DE) [3]. Although diagnostic laparoscopy may still be considered the gold standard in the diagnosis of endometriosis by many, as it allows direct visualization of the lesions, numerous studies have demonstrated the importance of transvaginal ultrasound scan (TVS) as a first-line imaging tool in the accurate diagnosis and pre-operative staging of endometriosis [4-9]. Despite this, gynecologists still appear to have poor awareness of TVS's role in the assessment of women with known or suspected endometriosis [10].

We designed an online survey that was distributed to gynecologists (members of the Hellenic Society of Gynecological Endoscopy, HSGE) practicing in Greece, aiming to check their views on the role of TVS in general, in the diagnosis and assessment of different phenotypes and locations of endometriosis, as well as their own clinical experience with using TVS for the diagnosis of endometriosis of the same phenotypes and locations. We also included questions on the need for specialized TVS training in the diagnosis and assessment of endometriosis. Then we checked if there was an alignment between the clinicians' views and own clinical practice for the diagnosis of endometrioma and all DE locations. Finally, we investigated a possible association between the participants' professional status, years of experience, and the number of TVSs per year and their answers.

## Materials and methods

Survey design and distribution

We designed an online survey on the website SurveyPlanet (app.surveyplanet.com) in December 2022. The questionnaire was designed initially by one of the authors (GG), and all questions were subsequently reviewed and agreed upon by all authors. The questionnaire comprised three main parts (see Appendix 1). The first part (questions 1-3) involved questions regarding the participants' background (professional status, years of experience post-residency, number of TVSs performed per year). The second part (questions 4-18) involved questions on the diagnosis of ovarian endometrioma and various locations of DE: bowel, bladder, ureter, parametrium, uterosacral ligaments (USLs), and rectovaginal septum/posterior vaginal vault. For each of those locations, the participants were first asked about their views on the diagnostic potential of TVS in general and then about their own ability to diagnose endometriosis in those locations using TVS. Possible answers included "always", "most of the time", "sometimes", "rarely", "never", and "not sure". The third part (questions 19-22) included questions about the role of TVS in the investigation of women with possible endometriosis, preoperative assessment of women with known endometriosis, as well as training. For all questions, only one answer was possible. Answering all questions was mandatory in order to complete the questionnaire.

The size of our availability sample was merely based on the number of participants that responded to our invitation and answered all questions of our online survey (rather than us calculating the necessary sample size in advance). An initial invitation by email to all members of the HSGE was sent in December 2022, and a reminder email was sent three weeks later. All HSGE members are clinicians post-residency. The online survey was closed in January 2023 and remained active for two months in total. The survey was distributed to members of HSGE, following relevant permission from the board of directors of the HSGE. The results were shared with and validated by the HSGE board of directors.

Aims of survey and statistical analysis

Our primary aim was to check and document the participants' beliefs on the role of TVS in the diagnosis of endometrioma and DE, their own ability to diagnose endometrioma and DE using TVS, and their views on the need for relevant specialized training. Based on the participants' answers, we present the descriptive statistics for all 22 questions. All variables resulting from answers are categorical. We aimed to answer the following research questions. The first question is about an association between the participants' belief of the ability of TVS to diagnose endometrioma and DE in various locations and their own clinical practice (or else, is belief aligned with clinical practice). In order to answer this, we used contingency tables (crosstabulation) and performed the chi-square test of independence. Since the sample is not large, the significance (p-value) of the chi-square test is estimated exactly and not asymptotically. A p-value less than 0.05 is considered significant, i.e., it shows that there is an association between the two answers. Since the categorical variables (belief-practice) are also ordinal, we calculated the Spearman correlation coefficient by taking values in the interval [-1,+1]. A value close to +1 shows a positive correlation, i.e., answers to both questions are aligned. The second research question was about the difference in answers to questions 4-22 (the second and third parts) according to the participants' professional status, years of experience (post-residency), and number of TVSs performed per year. Again, we used contingency tables and a chi-square test with an exact estimation of the significance. A p-value <0.05 shows a significant association.

## Results

**Figure 1:**
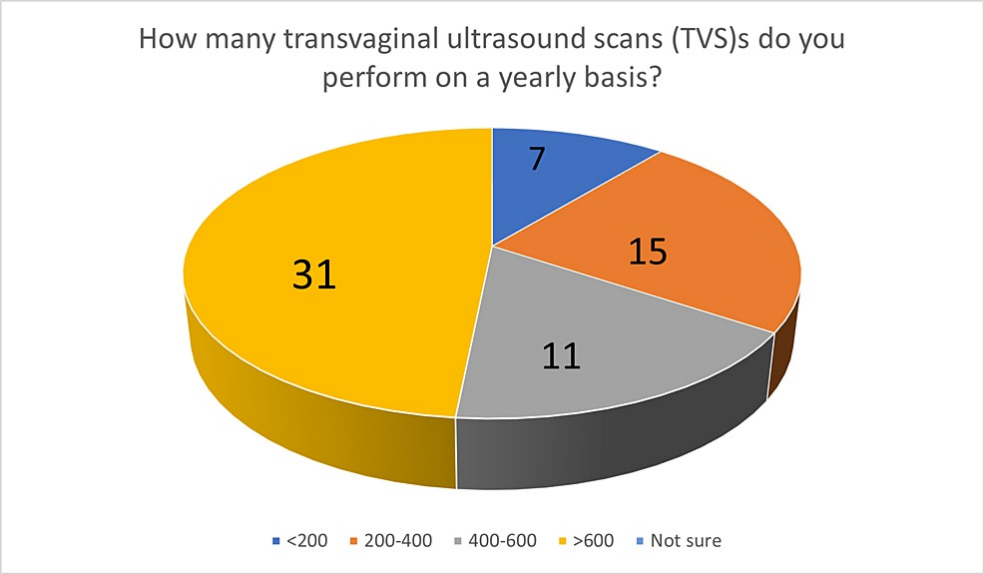
The number of transvaginal ultrasound scans performed by participants

We collected answers from 64 participants following an invitation (by e-mail) to 294 members of the HSGE. Each participant answered all questions of the questionnaire. Regarding the first part, the participants' professional status was as follows: 52 participants (81.25%) worked in the private sector, six (9.37%) were consultants in the Greek National Health Service, and six (9.37%) were academics. Regarding their clinical experience post-residency, 45 participants (70.31%) had 11 or more years of experience. All participants performed TVS on a routine basis. When asked to specify the average number of TVSs they perform on a yearly basis (Figure 1), 42 participants (65.62%) answered that they perform more than 400 TVSs per year.

**Figure 2:**
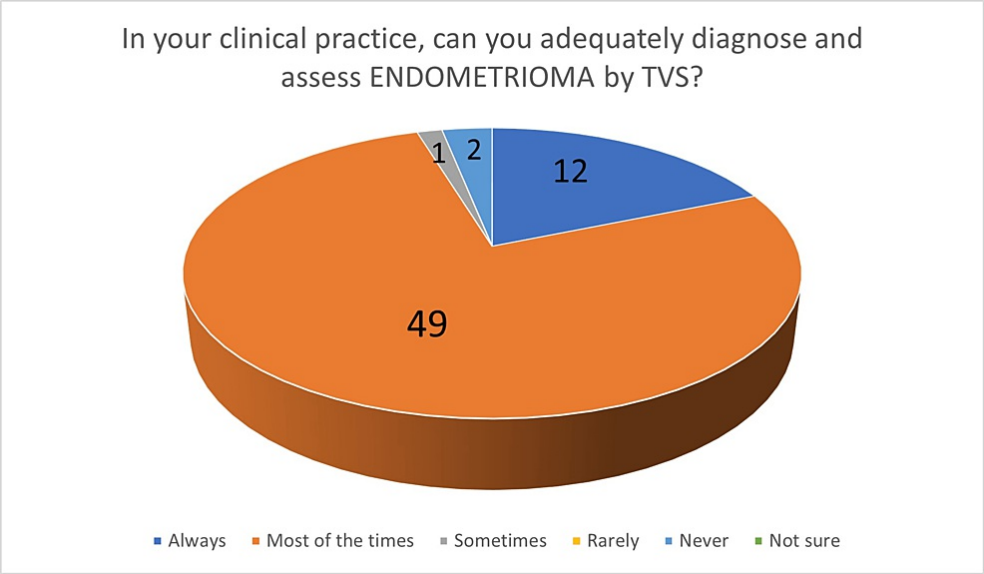
The number of answers on adequate diagnosis and assessment of endometrioma by TVS TVS - transvaginal ultrasound scan

**Figure 3:**
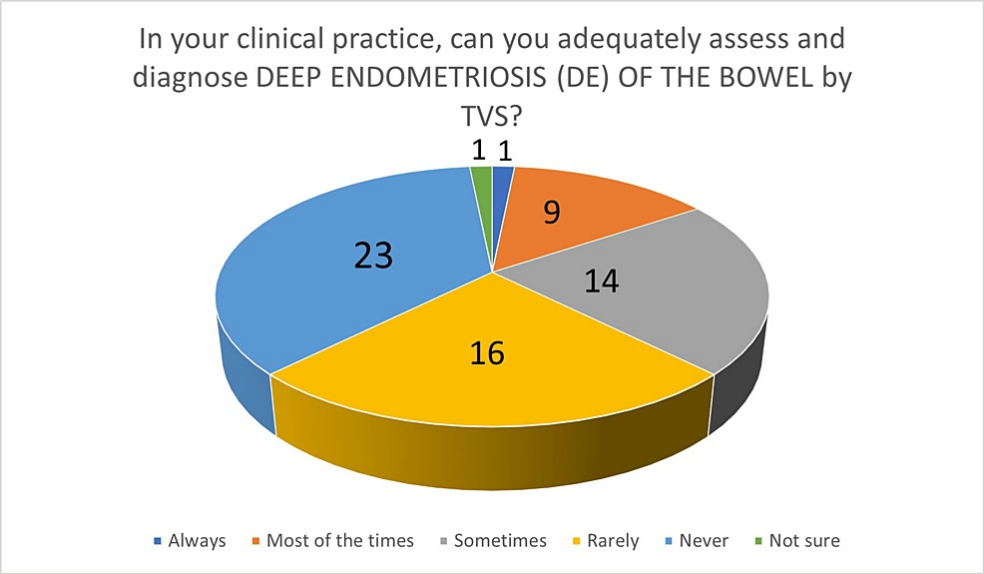
The number of answers on adequate assessment and diagnosis of deep endometriosis of the bowel by TVS TVS - transvaginal ultrasound scan

**Figure 4:**
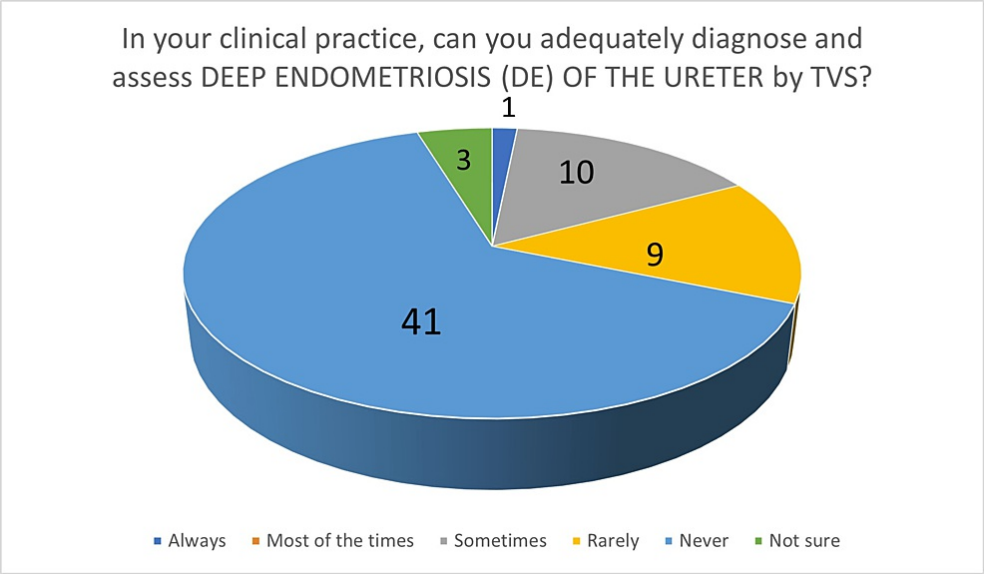
The number of answers on adequate diagnosis and assessment of deep endometriosis of the ureter by TVS TVS - transvaginal ultrasound scan

**Figure 5:**
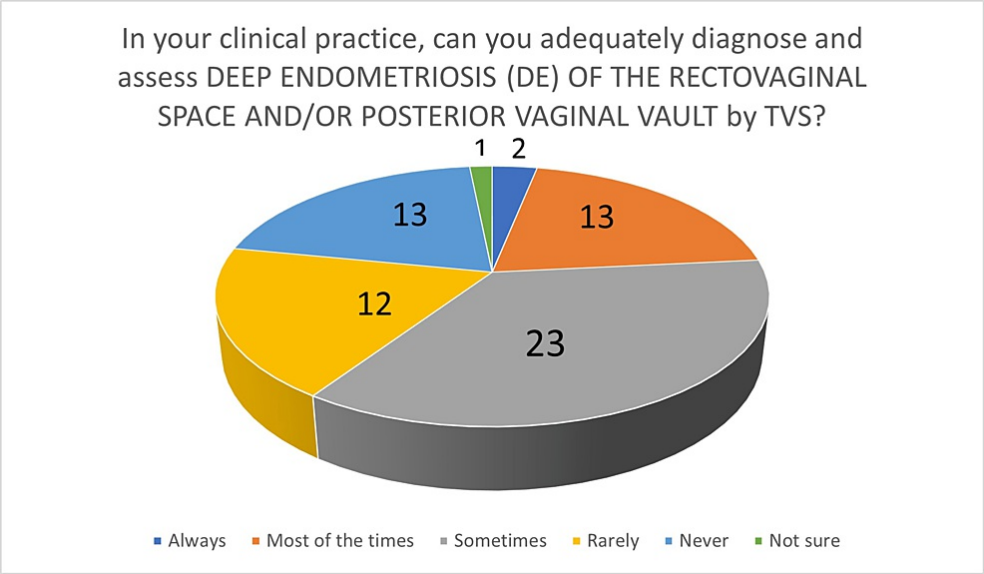
The number of answers on adequate diagnosis and assessment of deep endometriosis of the rectovaginal space and/or posterior vaginal vault by TVS TVS - transvaginal ultrasound scan

In the second part, participants were initially asked about the diagnosis of endometriomas by TVS: 60 participants (93.75%) felt that endometrioma can be diagnosed by TVS "always" or "most of the time". When asked about their own ability to diagnose endometrioma by TVS, 61 participants (95.31%) answered that they can confidently diagnose endometrioma by TVS "always" or "most of the time" (Figure 2). Regarding TVS performance in the diagnosis of DE of the bowel, 24 participants (37.5%) felt that TVS can "never" or "rarely" diagnose bowel DIE. When asked about their own clinical experience, 39 participants (60.94%) answered that they can "never" or "rarely" diagnose bowel DE by TVS (Figure 3). Participants were then asked about bladder endometriosis diagnosis by TVS: 23 gynecologists (37.94%) answered that bladder endometriosis can "never" or "rarely" be diagnosed by TVS. However, 37 participants (57.81%) answered that, in their own practice, they can "never" or "rarely" diagnose bladder endometriosis by TVS. Ureteric DE is generally considered to be a challenging diagnosis by TVS, and the results of our survey support this. Forty participants (62.5%) answered that ureteric DE can "never" or "rarely" be diagnosed by TVS, whereas, in their clinical practice, 50 participants (78.13%) felt that they can "never" or "rarely" diagnose ureteric DE by TVS (Figure 4). Parametrial DE can "never" or "rarely" be diagnosed by TVS, according to 24 of our participants (37.5%). In their own clinical practice, 35 participants (54.69%) could "never" or only "rarely" diagnose parametrial DE. Regarding DE of the USLs, 35 gynecologists (54.69%) answered that they can at least "sometimes" be diagnosed by TVS, whereas, when asked about their own clinical practice, 29 participants (45.31%) said that they are capable of at least "sometimes" diagnose DE of the USLs by TVS. Forty-seven participants (73.44%) believe that TVS can diagnose DE of the rectovaginal septum/posterior vaginal vault at least "sometimes" and 38 participants (59.38%), in their own clinical practice, can diagnose DE of the rectovaginal septum/posterior vaginal vault at least "sometimes" (Figure 5).

**Figure 6:**
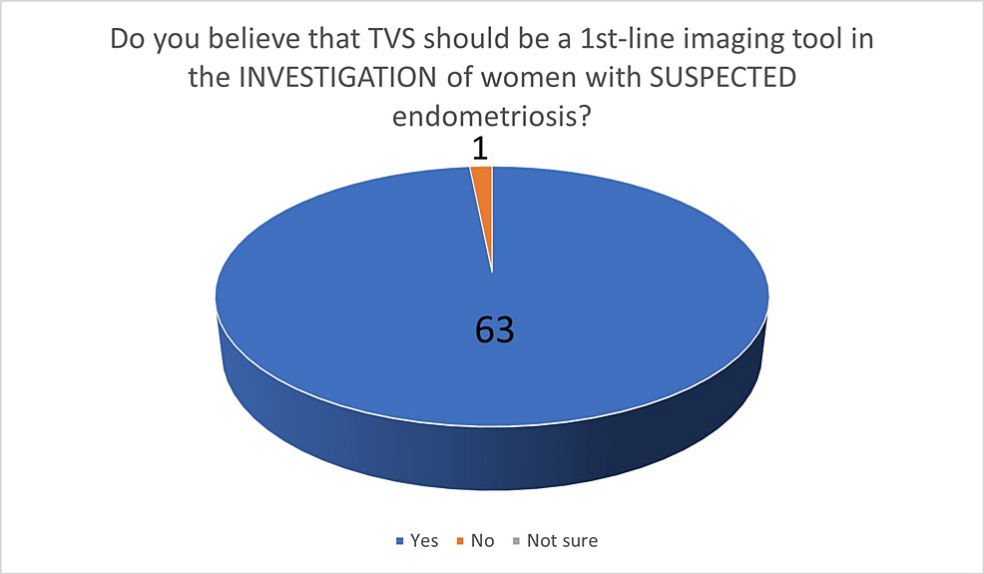
The number of answers on the belief that TVS should be the first-line imaging tool in the investigation of suspected endometriosis TVS - transvaginal ultrasound scan

**Figure 7:**
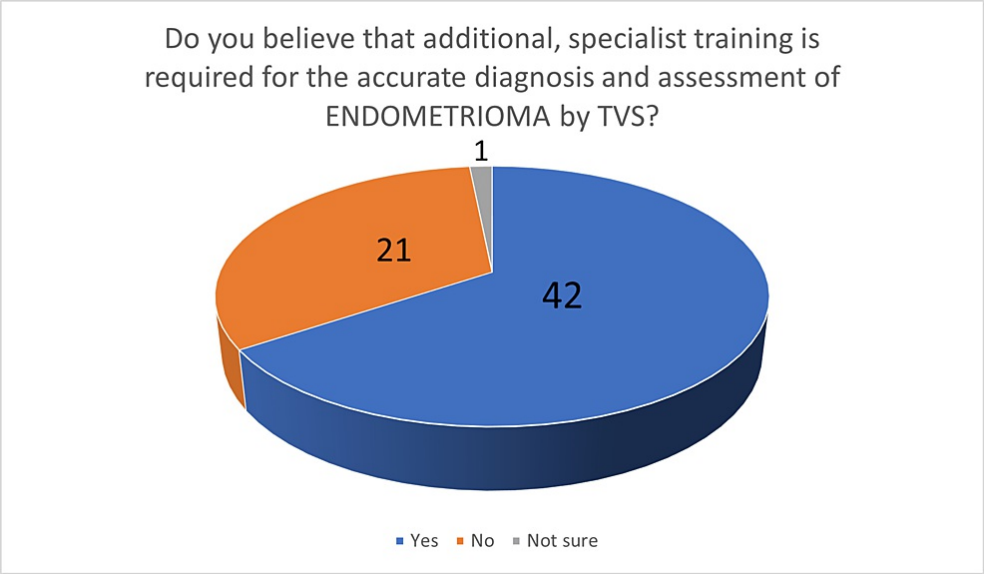
The number of answers on the belief that additional, specialized training is required for the accurate diagnosis and assessment of endometrioma by TVS TVS - transvaginal ultrasound scan

**Figure 8:**
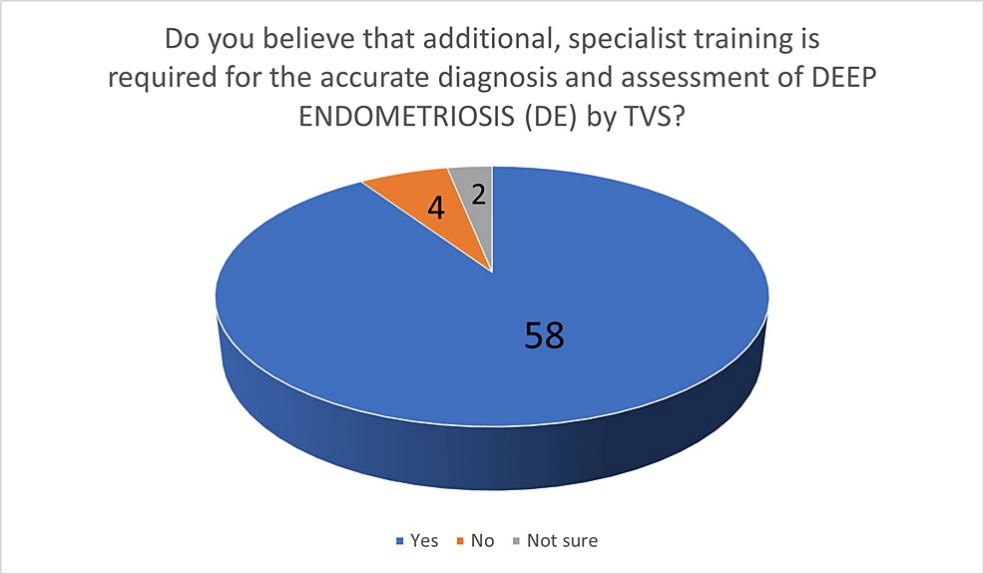
The number of answers on the belief that additional, specialized training is required for the accurate diagnosis and assessment of deep endometriosis by TVS TVS - transvaginal ultrasound scan

In the third part, 63 participants (98.44%) felt that TVS should be the first-line investigation in the investigation of women with suspected endometriosis (Figure 6). When asked about the preoperative staging of endometriosis in women with known endometriosis, 51 gynecologists (79.69%) stated that TVS should be considered as the first-line investigation. The last two questions were related to training in TVS. Participants were asked if they believed that additional, specialized training in TVS is required for the accurate diagnosis of endometrioma: 42 participants (65.62%) stated that additional, specialized training is required (Figure 7). When the same question was asked for the diagnosis of DE, 58 participants (90.62%) stated that additional, specialized training is necessary (Figure 8).

There was alignment (statistically significant correlation; all Spearman coefficients were higher than 0.7 with p<0.001) between the clinicians' beliefs and own clinical practice, both for the diagnosis of endometrioma as well as for all the aforementioned locations of DE. We did not identify an association between the clinicians' professional status or years of experience and their answers to questions 4-22. The only statistically significant association was the one between the number of TVSs per year and the clinician's ability to diagnose bowel DE by TVS in their clinical practice (p=0.008). It is worth noting that more than half (51.6%) of clinicians performing more than 600 TVSs per year answered that they can "never" diagnose bowel DE in their practice.

## Discussion

Accurate diagnosis of DE by TVS may be considered challenging by many gynecologists. Assessment of OMAs by TVS is generally considered more straightforward, whereas the value of TVS in diagnosing SUP is limited. The International Deep Endometriosis Analysis (IDEA) group reached a consensus on the terms and definitions that should be used in the diagnosis and assessment of different phenotypes of endometriosis and provided a structured approach for the investigation of women with endometriosis by TVS [11]. However, in an international survey of gynecologists by Leonardi et al. [10], the belief that TVS can diagnose bowel and USL DE was around 50%, demonstrating that the latest research in the field of TVS diagnosis of endometriosis has not reached many gynecologists. The National Institute for Health and Care Excellence (NICE) 2017 clinical guidance on endometriosis advises considering TVS in order to "identify endometriomas and deep endometriosis involving the bowel, bladder or ureter" [12]. The American College of Obstetrics and Gynecology (ACOG) 2010 Practice Bulletin stated that DE of the recto-vaginal septum (RVS) or rectum can be assessed by TVS [13]. The reported accuracies of TVS in the diagnosis of DE differ between studies [14-16]. There exist studies on the learning curve of TVS for the diagnosis of DE [17-19]. The issue of specialized training for the TVS diagnosis of endometriosis is an important one. In a study by Piessens et al. [20], following one week of specialized TVS training in DE diagnosis, competency could be achieved within 40 cases, allowing diagnosis of DE with similar diagnostic accuracy as highly specialized centers.

We focused our attention on the diagnosis of OMAs and various locations of DE by TVS. With the exception of endometriomas, for all locations of DE, the participant's views on the potential of TVS to diagnose DE were higher than their own diagnostic ability. Almost half of our participants were more than 15 years post-completion of their residency, and almost half of them performed more than 600 TVSs annually. This demonstrates that the majority of them had considerable experience in TVS. The participants had good confidence in their own ability, as well as the potential of TVS in general, to diagnose ovarian endometrioma. The results were markedly different in cases of DE. Of all DE locations, participants felt that ureteric DE is the most challenging to diagnose by TVS, scoring the highest percentage of "never" or "rarely", both in their views on the potential of TVS in general, as well as their own clinical experience. In contrast, they felt that TVS diagnosis of DE in the RVS/posterior vaginal vault is the easiest, with the highest percentage of "always", "most of the time", and "sometimes" answers, both in the ability of TVS to diagnose it in general, as well as in their own hands. With the exception of DE of the RVS/posterior vaginal vault, for all other DE locations, more than 50% of participants felt that they can "rarely" or "never" diagnose it by TVS in their own clinical practice.

We identify a strong statistically significant correlation between the clinicians' belief of TVS's ability and their own clinical practice, both for endometrioma as well as all DE locations. We feel that this is a reflection of the clinicians' general skepticism over the true role and value of TVS in the diagnosis and assessment of DE in general, in spite of the existing literature [4-9]. Other than an association between the number of TVS per year and diagnosis of bowel DE in clinical practice, we did not identify a significant association between the participants' professional role, years of experience, or number of TVSs per year and answers to all other questions. That is to say, their level of seniority or experience in TVS did not significantly impact their belief or clinical ability to diagnose DE by TVS. Indeed, as stated previously, more than half of participants performing more than 600 TVSs per year responded that they can "never" diagnose bowel DE by TVS. This demonstrates that diagnosis of endometriosis by TVS does not simply require experience and regular exposure to ultrasound but, rather, is an advanced, specialist skill that needs to be mastered through appropriate training and supervision.

There is a growing body of evidence suggesting that TVS should be a first-line tool in the investigation of women with endometriosis [5,9]. This was also reflected in our survey, as 63 out of 64 gynecologists (98.44%) that took part in our survey expressed the same opinion. As regards pre-operative staging of women with known endometriosis, the literature suggests that both TVS and MRI are valuable diagnostic modalities [21-24]. Almost eight out of 10 participants (79.69%) felt that TVS should be the first line in the pre-operative staging of women with known endometriosis.

Moving to the question about additional, specialized training, participants were asked separately about the diagnosis of endometrioma and DE: interestingly, although the majority of participants felt confident in their ability to diagnose endometrioma by TVS, 42 of them (65.6%) stated that additional, specialized training is required. When asked about the diagnosis of DE, 58 of them (90.6)% felt that additional, specialized training is required. In the topic of ultrasound training, ultrasound curricula differ between different regions [25]. Gynecological scanning in Greece is generally performed by gynecologists (neither radiologists nor sonographers) who may have different training backgrounds and variable levels of experience and expertise. In Greece, there is a six-month post-residency training program in advanced TVS for general gynecology, but not focused on endometriosis. 

We need to recognize some weaknesses of our study. Firstly, the small size of our available sample. We intentionally only sent the invitation to the survey to members of the HSGE. Our rationale for sending invitations to members of the HSGE (rather than members of the Hellenic Society of Obstetrics and Gynecology, HSOG) was that HSGE members were more likely to be colleagues with experience and interest in the diagnosis and/or management of endometriosis. Another possible weakness is the fact that, when asked about their own clinical practice, participants were not externally assessed by an expert on TVS diagnosis of endometriosis but, rather, expressed their personal opinions on their own ability. We also need to acknowledge that participants, both throughout their training, but also in their daily clinical practice, would have access to ultrasound machines of different resolution capabilities, and this is likely to have played a role in their beliefs and own diagnostic ability. We also need to elaborate on our decision to use bowel DE rather than specifying the exact location of the DE nodule in the bowel (low-mid rectum, high rectum, sigmoid, or higher). Albeit we recognize that the exact location of the DE bowel nodule is likely to affect the ability of the TVS operator to accurately diagnose and describe the nodule, we intentionally kept things simple so as to avoid putting the participants off by a large number of questions. Lastly, we need to comment on the possible answers to the second part of our survey's questions: although we recognize that "always" and "never" may be unrealistic, we felt that, by giving our reviewers those options, we would empower them to draw a clearer picture of their views and personal clinical experience for each location of endometriosis. In the future, similar surveys, but with a larger number of participants, both in Greece as well as internationally, may be useful in confirming or refuting the findings of our survey.

## Conclusions

The results of our survey suggest that, in contrast with the emerging view that TVS plays a key role in the diagnosis and assessment of DE, our participants overall still have low expectations from TVS in diagnosing DE. This is even more pronounced when asked about their own ability to diagnose DE by TVS. With the exception of bowel DE, answers to all other questions did not differ significantly based on the participants' professional role, years of experience, or number of TVs performed per year. This reflects an overall delayed adoption of TVS as a first-line tool in diagnosing endometriosis. Participants felt more confident in diagnosing endometrioma by TVS. The fact that nine out of 10 participants felt that additional, specialized training is required for the diagnosis of DE by TVS should, in our opinion, form the basis of discussions, within relevant bodies, on the possibility of this prospect.

## Appendices

**Table 1:** List of questions in the questionnaire

#	Question
Question 1	What is your current professional role?
Question 2	For how many years have you been practicing as a trained gynecologist?
Question 3	In your practice, do you perform transvaginal ultrasound (TVS) on a regular basis?
Question 4	How many TVSs do you perform on a yearly basis?
Question 5	Do you believe that ENDOMETRIOMAS can be adequately diagnosed and assessed by TVS?
Question 6	In your clinical practice, can you adequately diagnose and assess ENDOMETRIOMA by TVS?
Question 7	Do you believe that DEEP ENDOMETRIOSIS (DE) OF THE BOWEL can be adequately diagnosed and assessed by TVS?
Question 8	In your clinical practice, can you adequately assess and diagnose DEEP ENDOMETRIOSIS (DE) OF THE BOWEL by TVS?
Question 9	Do you believe that DEEP ENDOMETRIOSIS (DE) OF THE URINARY BLADDER can be adequately diagnosed and assessed by TVS?
Question 10	In your clinical practice, can you adequately diagnose and assess DEEP INFILTRATING ENDOMETRIOSIS (DIE) OF THE URINARY BLADDER by TVS?
Question 11	Do you believe that DEEP ENDOMETRIOSIS (DE) OF THE URETER can be adequately diagnosed and assessed by TVS?
Question 12	In your clinical practice, can you adequately diagnose and assess DEEP ENDOMETRIOSIS (DE) OF THE URETER by TVS?
Question 13	Do you believe that DEEP ENDOMETRIOSIS (DE) OF THE PARAMETRIUM can be adequately diagnosed and assessed by TVS?
Question 14	In your clinical practice, can you adequately diagnose and assess DEEP ENDOMETRIOSIS (DE) OF THE PARAMETRIUM by TVS?
Question 15	Do you believe that DEEP ENDOMETRIOSIS (DE) OF THE UTEROSACRAL LIGAMENTS can be adequately diagnosed and assessed by TVS?
Question 16	In your clinical practice, can you adequately diagnose and assess DEEP ENDOMETRIOSIS (DE) OF THE UTEROSACRAL LIGAMENTS by TVS?
Question 17	Do you believe that DEEP ENDOMETRIOSIS (DE) OF THE RECTOVAGINAL SPACE AND/OR POSTERIOR VAGINAL VAULT can be adequately diagnosed and assessed by TVS?
Question 18	In your clinical practice, can you adequately diagnose and assess DEEP ENDOMETRIOSIS (DE) OF THE RECTOVAGINAL SPACE AND/OR POSTERIOR VAGINAL VAULT by TVS?
Question 19	Do you believe that TVS should be a 1st-line imaging tool in the INVESTIGATION of women with SUSPECTED endometriosis?
Question 20	Do you believe that TVS should be a 1st line imaging tool in the PRE-OPERATIVE ASSESSMENT AND STAGING of women with KNOWN endometriosis?
Question 21	Do you believe that additional, specialist training is required for the accurate diagnosis and assessment of ENDOMETRIOMA by TVS?
Question 22	Do you believe that additional, specialist training is required for the accurate diagnosis and assessment of DEEP ENDOMETRIOSIS (DE) by TVS?
